# Bioactive Polysaccharides from *Gracilaria lemaneiformis*: Preparation, Structures, and Therapeutic Insights

**DOI:** 10.3390/foods13172782

**Published:** 2024-08-31

**Authors:** Min Wang, Zhen Zhu, Xiaocheng Wu, Kitleong Cheong, Xiaohua Li, Wanli Yu, Yinlin Yao, Jiang Wu, Zhanhui Cao

**Affiliations:** 1 College of Coastal Agriculture Sciences, Guangdong Ocean University, Zhanjiang 524088, China; wangmin@gdou.edu.cn (M.W.);; 2 College of Food Science and Technology, Guangdong Ocean University, Zhanjiang 524088, China; klcheong@gdou.edu.cn

**Keywords:** *Gracilaria lemaneiformis*, polysaccharides, extraction, biological activities, gut microbiota

## Abstract

*Gracilaria lamaneiformis*, a red seaweed, is an abundant source of bioactive polysaccharides with significant health-promoting properties. Nevertheless, the broad application of *G. lamaneiformis* in the nutraceutical and pharmaceutical sectors remains constrained due to the absence of comprehensive data. This review provides a detailed examination of the preparation methods, structural characteristics, and biological activities of *G. lamaneiformis* polysaccharides (GLPs). We explore both conventional and advanced extraction techniques, highlighting the efficiency and yield improvements achieved through methods such as microwave-, ultrasonic-, and enzyme-assisted extraction. The structural elucidation of GLPs using modern analytical techniques, including high-performance liquid chromatography, gas chromatography, and nuclear magnetic resonance spectroscopy, is discussed, providing comprehensive insights into their molecular composition and configuration. Furthermore, we critically evaluate the diverse biological activities of GLPs, including their antioxidant, anti-inflammatory, antitumor, and gut microbiota modulation properties. This review underscores the therapeutic potential of GLPs and suggests future research directions to fully harness their health benefits.

## 1. Introduction

The growing interest in marine resources for bioactive compounds has led to the exploration of various seaweeds [1,2], including *Gracilaria lamaneiformis*. This red algae species is widely distributed in coastal regions and has been traditionally valued in different cultures for its nutritional and medicinal benefits [3]. In China, the cultivation of *G. lamaneiformis* is primarily driven by its economic and environmental advantages. As a fast-growing seaweed, it is often used in integrated multi-trophic aquaculture systems to mitigate the environmental impact of fish and shrimp farming by absorbing excess nutrients, thereby improving water quality and the sustainability of aquaculture operations [4,5]. Besides its environmental benefits, *G. lamaneiformis* is a valuable resource in the food and nutraceutical industries due to its abundance of bioactive components, particularly polysaccharides [6].

*G. lamaneiformis* polysaccharides (GLPs) have garnered significant interest due to their unique structural characteristics and biological functions. The processes of extracting and purifying GLPs are crucial as they affect yield, purity, and biological efficacy. Traditional thermal extraction methods are commonly employed but are often inefficient and time-consuming [7]. To overcome these limitations, advanced techniques such as ultrasound-assisted extraction (UAE), microwave-assisted extraction (MAE), and enzyme-assisted extraction (EAE) have been developed [8]. These methods aim to enhance extraction efficiency, preserve bioactivity, and increase the overall yield of polysaccharides. The structural characterization of GLPs involves analyzing their monosaccharide composition, molecular weight, and structural configuration. Techniques like high-performance liquid chromatography (HPLC), gas chromatography (GC), and nuclear magnetic resonance (NMR) are widely used for this purpose [9]. These analytical methods provide detailed insights into the molecular structure of GLPs, which is crucial for understanding their biological activities. GLPs are primarily sulfated galactans, known for their diverse biological activities, including antioxidant, immunomodulatory, antitumor, and intestinal effects [10,11,12]. Understanding these structural features is essential for utilizing the therapeutic potential of GLPs.

This review aimed to deliver a thorough examination of the preparation, structural features, and biological activities of GLPs. We explored a range of extraction methods, including both traditional and innovative techniques. The structural characterization of GLPs was outlined using contemporary analytical methods, providing insights into their molecular composition and configuration. Additionally, we critically assessed the biological activities of GLPs, focusing on their potential therapeutic applications in health and disease management. This review underscores the notable advancements in GLP research and suggests future pathways for their development and application.

## 2. Extraction of GLPs

**Figure 1:**
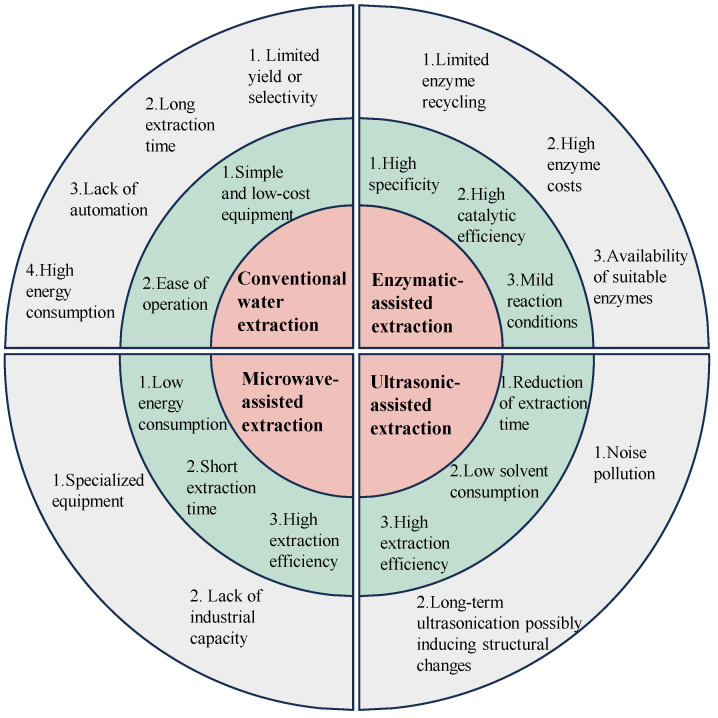
The pros and cons of the conventional extraction method and advanced extraction techniques, including microwave-assisted, ultrasonic-assisted, and enzymatic-assisted extraction. Red indicates the extraction methods, green highlights the advantages of each extraction method, and grey points out the disadvantages of each extraction method.

The extraction of GLPs is essential for their use in various industrial and health-related applications. Different extraction techniques, each with its own advantages and limitations, are summarized in Figure 1. The objective of extraction is to break down the cell wall and solubilize the polysaccharides in aqueous extract. The choice of extraction method, along with its unique mechanism and operating conditions, significantly affects the yield, purity, and bioactivity of the extracted polysaccharides. This section provides an overview of commonly used techniques, including traditional, EAE, MAE, and UAE methods. Understanding and optimizing extraction parameters can greatly enhance the process. The most influential factors are extraction temperature and time, which crucially affect the solubility of polysaccharides [13]. Higher temperatures generally improve extraction efficiency by enhancing solvent penetration, reducing viscosity, and increasing the dissolution of polysaccharides into the solvent [14]. However, excessively high temperatures can degrade thermolabile polysaccharides, leading to a loss of bioactivity and structural integrity [15]. Therefore, it is essential to balance these factors to maximize yield while preserving the quality of the polysaccharides.

### 2.1. Traditional Extraction Method

The traditional extraction of polysaccharides from *G. lemaneiformis* typically involves a straightforward and widely used hot water extraction process. In this method, dried and ground algae are submerged in hot water at temperatures ranging from 90 °C to 100 °C for an extended period. For example, *G. lemaneiformis* powder is soaked and extracted using hot water at 90 °C with a solid-to-liquid ratio of 1:45 for a duration of 4 h [16]. Acid solutions, such as citric acid (pH 2.0) at a solid-to-liquid ratio of 1:50 (*w*/*v*) at 100 °C for 3 h, are also used in extraction [17]. Additionally, alkali solutions are employed; semi-dried pieces of *G. lemaneiformis* are soaked in alkali solution (0.3 mol/L NaOH) at 25 °C for 2 h before being extracted with cold water three times, each extraction lasting for 2 h [12]. The heat in the extraction process facilitates the breakdown of cell walls and the extracting polysaccharides into the water. This method is advantageous due to its simplicity, cost-effectiveness, and minimal need for specialized equipment, making it particularly suitable for large-scale operations [18]. However, hot water extraction has notable limitations. It is time-consuming and may not efficiently extract all polysaccharides present in the biomass [19]. Additionally, the lack of specificity means other soluble components may be co-extracted, necessitating further purification steps. Despite these limitations, hot water extraction remains a foundational technique in the initial stages of polysaccharide research and production.

### 2.2. Microwave-Assisted Extraction Method

MAE is an advanced technique that utilizes microwave radiation to improve the extraction of polysaccharides from *G. lemaneiformis*. The principle behind MAE involves using microwaves to heat the solvent and biomass simultaneously. Microwaves cause rapid oscillation of polar molecules, generating heat through friction and dielectric heating [20]. This localized heating disrupts cell walls more efficiently than conventional methods, facilitating the release of polysaccharides into the solvent [21]. Process parameters, such as microwave power, exposure time, and solvent type, are carefully controlled to optimize extraction efficiency and yield. MAE provides several benefits compared to traditional thermal extraction methods. It considerably shortens extraction time and reduces solvent usage, making the process eco-friendlier and more cost-effective. For example, comparing MAE with hot water extraction under the same liquid-to-solid ratio of 1:20 and extraction temperature of 70 °C, the MAE using a 500 W microwave yielded a higher polysaccharide content (9.6%) than hot water extraction (9.1%). Additionally, MAE required only 20 min, compared to 60 min for hot water extraction [22]. The rapid and uniform heating provided by microwaves minimizes the thermal degradation of sensitive polysaccharides, preserving their bioactivity and structural integrity and ensuring better reproducibility [23,24]. However, the initial setup costs for microwave extraction can be high due to the specialized equipment required. Additionally, scaling up MAE from the laboratory to an industrial scale can be challenging due to differences in microwave penetration and heating uniformity in larger volumes [25,26].

### 2.3. Ultrasonic-Assisted Extraction Method

UAE is a cutting-edge technique that employs ultrasonic waves to improve the extraction of polysaccharides from *G. lemaneiformis*. This method involves applying high-frequency sound waves to a mixture of solvent and biomass, inducing cavitation bubbles within the liquid [27]. These bubbles collapse rapidly, releasing intense localized energy that disrupts the algae’s cell walls [28]. This mechanical disruption facilitates the release of polysaccharides into the solvent. Parameters such as ultrasound frequency, power, and duration are carefully optimized to maximize extraction efficiency. Tang et al. utilized UAE to extract *G. lemaneiformis* polysaccharides using an ultrasonic device at 65 °C for 30 min [29]. Furthermore, a combined technique employing ultrasound and microwave-assisted extraction was applied, with extraction conditions set at 87 °C, ultrasonic power of 50 W, microwave power of 800 W, extraction duration of 31.7 min, and a solid-to-water ratio of 1.0:60.7, resulting in a polysaccharide yield of 34.8%, compared to 29.7% for hot water extraction [30]. UAE offers significant benefits over conventional methods. It shortens extraction time and solvent usage, enhancing cost-effectiveness and environmental sustainability. The cavitation effect ensures efficient disruption of cell walls and improves solvent penetration, leading to higher yields. Moreover, the lower temperatures used in UAE help to preserve the bioactive and structural stability of thermally sensitive polysaccharides. However, the initial investment in ultrasonic equipment may be prohibitive for smaller operations considering adopting this technology.

### 2.4. Enzymatic-Assisted Extraction Method

EAE leverages specific enzymes to dismantle the cell walls of *G. lemaneiformis*, facilitating the release of polysaccharides in a mild and efficient manner. This process involves cell-wall-degrading enzymes, such as cellulases, amylase, hemicellulases, and pectinases, which hydrolyze the complex carbohydrates and proteins that make up the algal cell walls [31,32]. The enzymatic action disrupts the structural integrity of the cells, allowing polysaccharides to be solubilized and extracted into the surrounding solvent [33]. Key extraction parameters include the type and concentration of enzymes, temperature, and pH to maximize enzymatic activity. For example, enzymatic extraction using a thermostable α-amylase from *Thermococcus* sp. HJ21 at a high temperature of 95 °C and pH 5 resulted in a yield of *G. lemaneiformis* polysaccharides reaching 49.15% on a dry weight [34]. Additionally, some reports indicate that extraction efficiency can be increased by adding enzymes to the initial hot water extract mixture, supplementing with 1% papain and 0.5% cellulase (*w*/*v*) and incubating at 60 °C for 2 h to obtain more polysaccharides [16]. Chen et al. reported that the H_2_O_2_-assisted cellulase extraction of agar from *G. lemaneiformis* preserves the sulfate content, achieving about 3.56%, which is higher than the 1.80% sulfate content obtained with traditional alkali-extracted agar [35]. The benefits of enzymatic extraction include its high specificity, targeting only cell wall components and minimizing the extraction of unwanted substances, thus improving the purity of the polysaccharides [36]. Additionally, enzymatic extraction operates under mild conditions, which helps preserve the bioactivity and structural integrity of the polysaccharides, making them suitable for sensitive applications such as pharmaceuticals and nutraceuticals. However, the cost of enzymes and the need for the careful control of process parameters can be seen as limitations [37].

### 2.5. Other Extraction Techniques

Liquid-phase pulsed discharge is a non-thermal technique developed based on electrical breakdown in water. This method generates UV light emission, radical species, high-amplitude shock waves, and cavitation, all of which promote the release of intracellular polysaccharides [38,39]. Ju and Xi reported the liquid-phase pulsed discharge extraction of *G. lemaneiformis* polysaccharides, using an electric field strength of 80 kV/cm and a flow velocity of 14 mL/min. Although the extraction yields were not significantly different—140.19 mg/g for hot water extraction and 147.94 mg/g for liquid-phase pulsed discharge extraction—the latter method offered notable advantages. Specifically, it required a shorter extraction time (79 min versus 300 min) and operated at a lower temperature (25 °C versus 60 °C) [40].

## 3. Purification of GLPs

**Figure 2:**
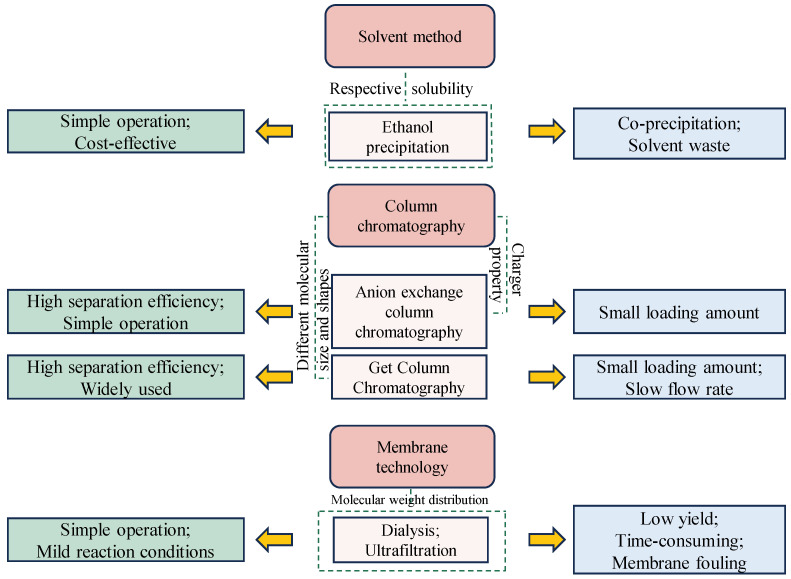
Summary of the advantages and disadvantages of different purification techniques for GLPs, including ethanol precipitation, column chromatography, and membrane technology.

Purifying GLPs is vital for producing high-quality, consistent polysaccharide preparations for research and industrial use. Effective purification methods are essential to remove impurities that might interfere with the structural analysis and biological evaluation of GLPs. Achieving high-purity polysaccharides is crucial for accurately determining structure–function relationships, which are key to understanding their biological activities and ensuring they meet the rigorous quality standards required for pharmaceuticals, nutraceuticals, and functional foods. A summary of the advantages and disadvantages of different purification techniques has been shown in Figure 2. Ethanol precipitation is a commonly employed technique for purifying GLPs due to its simplicity, cost-effectiveness, and efficiency in removing contaminants. This process involves adding ethanol to an aqueous solution of crude polysaccharide extract, resulting in the precipitation of polysaccharides while smaller molecules and impurities remain dissolved in the ethanol–water mixture [41,42]. Researchers typically adjust the ethanol concentration to between 30% and 80% to isolate GLP fractions [12,43]. However, ethanol precipitation has limitations, including the potential co-precipitation of low molecular weight impurities, which reduces the overall purity of the final product and necessitates additional purification steps [44,45]. Additionally, the requirement for large volumes of ethanol can be both costly and hazardous.

Membrane separation techniques use semi-permeable membranes to separate molecules by size, making them effective for purifying polysaccharides [46,47], including those from *G. lamaneiformis*. This method offers several benefits, such as low energy consumption, scalability, and the ability to operate at ambient temperatures. Membranes with a molecular weight cut-off of 1 kDa are typically used to dialyze and remove low molecular weight impurities from GLPs [48]. Dialysis membranes are commonly used to separate GLPs with varying molecular weights. Ultrafiltration is another specific membrane purification method that utilizes membranes with various pore sizes to purify polysaccharides of different molecular weights [49]. By choosing membranes with particular pore sizes, researchers can effectively separate polysaccharides according to their size. For instance, membranes with larger pores (e.g., 100 kDa) can retain high-molecular-weight polysaccharides while allowing smaller molecules and impurities to pass through. Conversely, membranes with smaller pores (e.g., 10 kDa) can isolate low-molecular-weight polysaccharides by excluding even smaller contaminants [50,51]. Membrane separation techniques are continuous processes, making them suitable for large-scale industrial applications [52]. However, membrane fouling, where impurities or the polysaccharides themselves block the membrane pores, can be a significant drawback, reducing efficiency and increasing maintenance requirements [53].

Chromatographic techniques like ion-exchange chromatography and size-exclusion chromatography are highly selective and efficient for purifying GLPs, resulting in highly purified fractions. Ion-exchange chromatography separates GLPs based on their charge properties [54,55]. By gradually adjusting the ionic strength or pH of the elution buffer, polysaccharides with different charge densities can be selectively eluted [56]. Neutral and acidic GLPs are purified using DEAE Sephadex A-50 resin, eluted with distilled water and 0.3–0.6 mol/L NaCl solution [30]. DEAE-52 and DEAE-Sepharose fast-flow column resins can also be used to obtain sulfated polysaccharides, eluted with 0.4 mol/L NaCl solution [57,58]. Size-exclusion chromatography or gel-filtration chromatography purifies GLPs based on their molecular size [59,60]. Using Sephadex G-100 size-exclusion chromatography, eluted with distilled water, results in high-purity homogeneous peaks for GLPs [61,62]. Despite their effectiveness in achieving high-purity GLPs, these chromatographic techniques present challenges such as high costs for purification materials and the time-consuming nature of the processes [63].

## 4. Structural Characterization of GLPs

Structural characterization is vital in studying GLPs as it provides insights into their functional properties, biological activities, and potential applications. This analysis includes determining the monosaccharide composition, glycosidic linkages, molecular weight, and chain conformation of the polysaccharides [64,65]. Chromatography methods like HPLC and GC, along with spectroscopy techniques such as Fourier transform infrared spectrometer, multi-angle laser light scattering (MALLS), and NMR, are essential for obtaining detailed structural characterization.

### 4.1. Monosaccharide Composition

Monosaccharide composition is a vital structural feature of GLPs, offering key insights into the types and proportions of monosaccharides present. To determine this composition, polysaccharides are initially hydrolyzed into their constituent monosaccharides. These monosaccharides are then separated and quantified using chromatographic techniques, such as HPLC or GC [66,67]. For example, polysaccharides from *G. lamaneiformis* are hydrolyzed with trifluoroacetic acid at 100 °C for 2 h. The resulting monosaccharide solution is subjected to PMP derivatization and analyzed by HPLC. This analysis revealed that GLPs are predominantly composed of galactose (82.76%), rhamnose (9.01%), mannose (6.25%), and glucose (1.97%) [68]. In another study, GLPs were hydrolyzed using 4 mol/L TFA at 105 °C for 2 h. The resulting hydrolyzed product was derivatized with hydroxylamine hydrochloride and pyridine, followed by acetylation with acetic anhydride. Subsequent analysis by GC revealed the monosaccharide composition of GLPs, showing molar ratios of 45.84% galactose, 35.17% glucose, 18.12% xylose, and 0.33% mannose [58].

The anhydrogalactose residues are one of the main monosaccharide components in GLPs. However, they are acid- abile and easily converted to galactose residues or 5-hydroxymethyl-furfural under strong acid hydrolysis conditions [69]. For this reason, a reductive hydrolysis using the methylmorpholine–borane complex under mild acidic conditions is employed, which is suitable for converting GLPs into monosaccharides. After hydrolysis into monosaccharide components, the resulting alditols are acetylated and then subsequently analyzed by GC [70]. The results showed that different red seaweed polysaccharides had varying monosaccharide compositions. *Porphyra haitanensis*, *Gracilaria blodgettii*, and *Gracilaria chouae* consisted of anhydrogalactose and galactose with molar ratios of 1:1.4, 1:1.5, and 1:1.6, respectively. In contrast, *G. lemaneiformis* and *Eucheuma galetinae* had relatively high amounts of galactose, with molar ratios of 1:3.0 and 1:3.1, respectively [71].

### 4.2. Molecular Weight

Determining the molecular weight of GLPs is typically conducted using HPLC coupled with a size-exclusion column. In this method, polysaccharide molecules are separated based on their size as they move through a column packed with porous beads. The molecular weight is calculated using a series of dextran standards with known molecular weights. A study determined that a low molecular weight GLP had an average molecular weight of 2.7 × 10^3^ Da using the HPLC dextran calibration method [72]. Additionally, HPLC has been used to investigate the impact of various extraction and degradation methods on the molecular weight of GLPs. Liao et al. utilized HPLC to detect GLPs that had molecular weightw of 1.2189 × 10^5^ Da, and their two degradation products, with molecular weights of 57.02 × 10^3^ Da and 14.29 × 10^3^ Da, respectively [73]. Chen et al. used HPLC to determine the molecular weight of GLPs. The GLPs extracted enzymatically had larger molecular weights of 1.936 × 10^6^ Da, compared to the alkali-extracted GLPs, which had molecular weights of 7.91 × 10^5^ Da [35].

High-performance size-exclusion chromatography coupled with MALLS is a sophisticated method for determining the molecular weight of GLPs without the need for dextran calibration. This technique not only provides precise and accurate molecular weight measurements but also determines the radius of gyration and intrinsic viscosity, giving a detailed understanding of the polysaccharide’s chain conformation. Using HPSEC-MALLS combined with a viscometer, Veeraperumal et al. found that the molecular weight and intrinsic viscosity of GLPs were 1.570 × 10^5^ Da and 133.94 mL/g, respectively. Furthermore, the exponent α of the Mark–Houwink equation [η] = kMw^α^ for GLPs in sodium nitrate solution was calculated to be 0.737, indicating that GLP has a flexible chain in investigated aqueous solution [57].

### 4.3. Glycosidic Linkages

The glycosidic linkages of GLPs involve identifying the specific connections between monosaccharide units in the polysaccharide chain. Methylation analysis, followed by gas chromatography–mass spectrometry (GC–MS), is commonly used to determine these linkage positions. For instance, Shi et al. used this method to confirm that GLP consists of linear linkages of 1,3-linked galactopyranose and 1,6-linked galactopyranose [30]. Similarly, Bajwa et al. found that *Gracilariopsis* sp. polysaccharides contained a significant amount of 3-linked galactopyranose (40%) and 4-linked anhydro-galactopyranose (27%) through methylation analysis [74].

NMR spectroscopy is a powerful tool for investigating detailed structural information about polysaccharides, including the sequence of monosaccharides and the types of glycosidic linkages present. Studies have shown that the chemical structure of GLPs is primarily composed of galactose. For example, Li et al. described GLPs as having a linear backbone of β-(1→3)- and α-(1→4)-linked galactopyranose residues and anhydro-galactose units [43]. Similarly, Wang et al. described a GLP backbone consisting of alternating α-(1→3)-galactopyranose and β-(1→4)-galactopyranose units [72]. Another study investigated the chemical structure of GLP using various NMR techniques, including ^1^H NMR, ^13^C NMR, COSY, HSQC, and HMBC, revealing that the structure consists mainly of glucose and galactose, specifically →4)-α-D-Glc*p*-(1→4)-α-D-Glc*p*-(1→4)-β-D-Gal*p*-(1→6)-α-D-Glc*p*-(1→6)-α-D-Glc*p*-(1→ [75].

## 5. Biological Activities of GLPs

### 5.1. Antioxidant Activities

GLPs possess a multitude of bioactive properties, with their antioxidant activity being one of the most reported. GLPs exhibit robust antioxidant activities through several well-documented mechanisms, primarily by scavenging free radicals—unstable molecules that cause significant cellular damage through oxidative stress [76,77]. The biological activities, related mechanisms of action, and structural information of GLPs are summarized in Table 1. Reactive oxygen species (ROS), including superoxide anions, hydroxyl radicals, and hydrogen peroxide, as well as reactive nitrogen species such as nitric oxide and peroxynitrite, are pivotal in the onset and progression of various diseases, including cancer, cardiovascular disorders, and neurodegenerative conditions [78,79]. GLPs can donate hydrogen atoms or electrons to these reactive species, neutralizing them and mitigating their harmful effects. Wang et al. demonstrated the radical scavenging activities of GLPs in vitro using assays such as the superoxide radical assay, hydroxyl radical assay, DPPH (2,2-diphenyl-1-picrylhydrazyl) radical scavenging assay, and reducing power assay [80]. In another study, the scavenging ability of GLPs showed an IC_50_ of 9.62 mg/mL for ABTS (2,2′-azinobis(3-ethylbenzothiazoline-6-sulfonic acid)) radicals, 23.85 mg/mL for DPPH, 4.97 mg/mL for ferric ion, and 3.56 mg/mL for superoxide radicals [81]. Additionally, in cell culture studies on human keratinocyte cells (HaCaTs) exposed to H_2_O_2_, a common inducer of oxidative stress, GLPs significantly reduced ROS levels and increased the expression of Nrf-2/Keap-1, a key transcription factor regulating antioxidant responses [81]. This radical scavenging capability of GLPs is crucial for maintaining cellular homeostasis and preventing oxidative damage to tissue and organ proteins, lipids, and nucleic acids.

In addition to directly neutralizing free radicals, GLPs enhance the body’s antioxidant defense systems by upregulating the activity of endogenous antioxidant enzymes, including superoxide dismutase (SOD), catalase (CAT), and glutathione peroxidase (GPx). SOD catalyzes the dismutation of superoxide radicals into oxygen and hydrogen peroxide, which CAT then breaks down into water and oxygen. GPx further reduces hydrogen peroxide to water, using glutathione as a substrate. By increasing the levels and activity of these enzymes, GLPs bolster the cellular antioxidant defense mechanism, enhancing the overall capacity to detoxify harmful ROS. Fang et al. investigated the antioxidant activity of GLPs in in vitro cell culture assays and reported that GLPs alleviated H_2_O_2_-induced oxidative injury in human fetal lung fibroblast 1 cells by protecting them from oxidative damage and increasing the activities of SOD, CAT, and GPx [82].

Aging and diabetes are strongly connected to oxidative stress, which occurs when there is a disparity between the production of ROS and the body’s ability to counteract them with antioxidant defenses [83,84]. Antioxidant enzyme activity typically declines with age and chronic hyperglycemia, exacerbating oxidative damage [85,86]. In vivo studies indicate that GLPs can mitigate oxidative stress related to both aging and diabetes. Zhang et al. found that GLPs markedly restored the levels of antioxidant enzymes—SOD, CAT, and GSH-Px—in the serum and brain of D-galactose-induced aging mice in a dose-dependent manner [87]. Similarly, in streptozotocin-induced diabetic mice, GLP treatment significantly increased the levels of SOD, GSH-Px, and CAT [88]. In addition, ultraviolet (UV) radiation in the environment can induce oxidative stress in living organisms. UV radiation, particularly UVA (320–400 nm) and UVB (280–320 nm) wavelengths, penetrates the skin and interacts with cellular components, generating ROS. In an in vitro assay, UVB-induced damage decreased intracellular levels of GSH and SOD in HaCaT cells, while GLP treatment increased these levels, demonstrating that GLPs protected the cells from UVB-induced damage [89].

### 5.2. Immuno-Modulatory Activity

The immune system is essential for overall health and well-being, serving as the body’s primary defense against pathogens and disease [90]. It consists of a complex network of cells, tissues, and organs that work together to detect and neutralize harmful invaders such as bacteria, viruses, fungi, and parasites [91]. GLPs have demonstrated significant immunomodulatory activities by activating macrophages. These cells are vital to the innate immune system, as they detect, engulf, and destroy pathogens and apoptotic cells, thereby enhancing the body’s immune responses [92]. Ren et al. reported that treatment with GLPs has been shown to increase the phagocytic capability of RAW264.7 cells and increase the production of nitric oxide and ROS. Additionally, GLPs stimulate the secretion of pro-inflammatory cytokines such as tumor necrosis factor-alpha (TNF-α) and interleukin-6 (IL-6), further amplifying the immune response [58]. Proinflammatory cytokines such as tumor necrosis factor-alpha (TNF-α), interleukin-1 beta (IL-1β), and interleukin-6 (IL-6) are produced by immune cells, including macrophages, dendritic cells, and T-lymphocytes, in response to pathogens or cellular damage [93,94]. These cytokines are crucial mediators of the immune response, promoting inflammation to defend against infections and injuries. However, the overproduction or prolonged presence of proinflammatory cytokines can lead to chronic inflammation, contributing to the progression of various inflammatory diseases such as rheumatoid arthritis, inflammatory bowel disease, and cardiovascular diseases [95,96]. Thus, maintaining a balanced cytokine response is critical for effective immune defense while preventing the detrimental effects of chronic inflammation. In a lipopolysaccharide-induced IEC-6 cell model, GLP co-culture with IEC-6 cells decreased the release and inhibited the gene expression of TNF-α, IL-6, and IL-1β, demonstrating significant anti-inflammatory effects [43,97].

Another mechanism of GLP’s immunomodulatory effect involves modulating lymphocyte proliferation and differentiation. Lymphocytes, including T cells and B cells, play pivotal roles in adaptive immunity [98]. In individuals with food allergies, the immune system mistakenly identifies harmless food proteins as threats [99], triggering the activation of T-helper 2 (Th2) cells. These cells release cytokines such as IL-4 and IL-13. Liu et al. investigated GLP’s potential to alleviate food allergies using tropomyosin-sensitized mice. They found that GLPs significantly reduced Th2-dependent tropomyosin-specific IgE and IgG1 serum levels and decreased the production of IL-4 and IL-13. The immunosuppressive mechanism may be related to a reduction in p38 MAPK activity, contributing to the mitigation of food allergy symptoms [11,100,101]. The oligosaccharide derived from GLPs has been demonstrated to modulate type 1 immunity by suppressing T cell activation, as evidenced by studies conducted both in vivo and in vitro. In vivo, this oligosaccharide reduced the production of interferon-gamma (IFN-γ) in mice immunized with ovalbumin. In vitro, using OT-II CD4^+^ T cells, the oligosaccharide inhibited mTOR activity, glycolysis, cell cycle progression, and DNA replication [102].

### 5.3. Anti-Tumor Activity

The significance of polysaccharides derived from natural resources in cancer research is notably accentuated by their low or non-toxicity, distinguishing them from many conventional chemotherapeutic agents [103,104]. GLPs exhibit anti-tumor activity primarily through significant immune modulation. They enhance immune cell activity by stimulating the proliferation and activation of immune cells such as T lymphocytes, natural killer cells, and macrophages [105]. Ji et al. investigated the antitumor effects of GLPs on Kunming mice transplanted with S180 tumor cells in the armpit of the right hind limbs. They found that GLPs suppressed the aggressive growth of solid S180 tumors by enhancing the proliferation of splenocytes, increasing the cytotoxic activity of natural killer cells, and elevating the serum cytokine levels of IL-2, IFN-γ, and TNF-α [106]. This cytokine modulation not only boosts overall immune surveillance and attack on tumor cells but also helps reduce the immunosuppressive environment often created by tumors [107].

Moreover, GLPs exhibit antitumor activity through the direct inhibition of tumor growth. Kang et al. reported that GLPs showed inhibitory effects on the growth of three cancer cell lines: human gastric cancer cell line MKN45, cervical carcinoma cell line HeLa, and non-small cell lung cancer cell line A549. Their transcriptome analysis demonstrated that GLPs regulated apoptosis, the cell cycle, nuclear division, and cell death-related genes [108]. Shi et al. also reported similar findings, demonstrating that GLPs inhibit the proliferation of various cancer cell lines in vitro. These include human breast cancer cell MCF-7, human cervical carcinoma cell HeLa, and the human hepatocellular carcinoma cell HepG2 [30]. Another study demonstrated that GLPs inhibit the proliferation of various cancer cell lines, including gastric cancer cell line MKN28, human lung cancer cell line A549, and mouse melanoma cell line B16. This suppression is associated with the increased expression of the Fas/FasL signaling pathway [62]. Fas (CD95) is a death receptor located on the cell surface, and its ligand, FasL, binds to it, triggering a cascade of events that lead to programmed cell death [109]. GLPs enhance the expression of both Fas and FasL or increase their interaction. This activation of the Fas/FasL pathway promotes the formation of the death-inducing signaling complex, which further facilitates apoptosis. Cai et al. demonstrated that GLPs effectively inhibit tumor growth in a mouse model bearing colon-26 carcinoma. This anti-tumor effect is primarily mediated through the induction of ferroptosis and the regulation of ferroptosis-related metabolic pathways [110]. Ferroptosis is a type of programmed cell death characterized by the accumulation of lipid peroxides, and the depletion of intracellular glutathione is crucial for the antitumor activity of investigated samples [111]. The study found that GLPs promote ferroptosis by inhibiting glutathione synthesis and disrupting the function of glutathione peroxidase 4, an enzyme essential for protecting cells from lipid peroxidation. Additionally, GLPs increase the levels of 4-hydroxy-2-nonenal, a marker of lipid peroxidation, further driving ferroptosis. This mechanism highlights the potential of GLPs as a therapeutic agent that leverages ferroptosis to enhance its efficacy in targeting cancer cells, offering a promising approach for cancer treatment [110].

### 5.4. Intestinal Health and Gut Microbiota

The gut microbiota is a diverse population of microorganisms residing in the gastrointestinal tract, and it is essential for maintaining intestinal health and overall well-being [112]. A healthy gut ensures efficient digestion and nutrient absorption, supports a robust immune system, and maintains the integrity of the gut barrier, preventing harmful pathogens and toxins from entering the bloodstream [113,114]. Disruptions in gut health can lead to various diseases. Conditions like inflammatory bowel disease (IBD), which encompasses ulcerative colitis and Crohn’s disease, involve the chronic inflammation of the gut lining, causing severe abdominal pain, diarrhea, and malnutrition [115,116]. Furthermore, gut dysbiosis has been linked to metabolic diseases such as obesity, type 2 diabetes, heart disorders, and non-alcoholic fatty liver disease [117,118,119]. Beyond intestinal and metabolic health, the gut microbiota also significantly influences mental health through the gut–brain axis, a bidirectional communication network connecting the central nervous system and the gastrointestinal tract [120]. Therefore, maintaining a balanced gut microbiota is essential for promoting intestinal health and preventing disease [121].

Prebiotics are non-digestible food components that positively impact the host by promoting the growth or activity of specific beneficial microorganisms in the colon [122,123]. GLPs can serve as prebiotics because they remain undigested in the upper gastrointestinal tract. Studies have shown that GLP resists digestion by simulated gastric conditions, including exposure to gastric juice containing pepsin, gastric lipase, and HCl at pH 3.0. Additionally, GLP is not digested by amylase or by components of intestinal juice such as pancreatin, trypsin, and bile salts [17]. These findings indicate that GLP remains intact in the upper gastrointestinal tract and may reach the large intestine, where it can be fermented and utilized by gut microbiota.

The intestinal flora consists of a diverse community of microorganisms, predominantly from four phyla: Firmicutes, Bacteroidetes, Actinobacteria, and Proteobacteria [124]. GLPs can be fermented by Bacteroidetes. An in vitro simulated human fecal fermentation assay showed that GLPs increased Bacteroidete’s relative abundance while decreasing that of Firmicutes [10]. Bacteroidetes utilize various carbohydrate-active enzymes (CAZymes) to break down complex polysaccharides. These include glycoside hydrolases, which cleave glycosidic bonds in polysaccharide chains [125]. CAZymes are categorized by their specific functions and substrate preferences, allowing Bacteroidetes to degrade a wide range of polysaccharides such as xylans, glucan, mannan, arabinans, and agarans [64,126]. It has been established that Bacteroides plebeius possesses an endo-type β-agarase, BpGH16A, which is part of the glycoside hydrolase (GH) family 16. This enzyme breaks β-1,4-glycosidic bonds in agarose, generating neoagarooligosaccharides. [127]. Co-culturing red seaweed polysaccharides with *Bacteroides thetaiotaomicron* revealed the presence of a GH16 family enzyme that catalyzes the hydrolysis of β-D-galactopyranose-(1→4)-α-L-galactopyranose-β-6-sulfate linkages [128]. Additionally, the bacterium *Aquimarina* sp. AD1, which belongs to the Bacteroidetes phylum, contains GH96 family enzymes with specific activity targeting the α-1,3 linkage in LAα1 → 3G6S of funoran [129]. Bacteroidetes engage in a synergistic approach to break down complex polysaccharides. For instance, *Bifidobacteria* and *Lactobacillus* further process oligosaccharides that Bacteroidetes initially degrade. This interspecies cooperation involves exchanging intermediate metabolites, enhancing the breakdown of dietary fibers and improving the efficiency of carbohydrate fermentation. This is why GLPs can increase the relative abundance of both *Bacteroidetes* spp. and *Bifidobacteria* spp., while reducing the relative abundance of *Escherichia* [10].

The gut microbiota breaks down GLPs into smaller carbohydrates and monosaccharides, leading to the production of short-chain fatty acids (SCFAs) [130,131]. The main SCFAs are acetate, propionate, and butyrate, each contributing uniquely to gut health and overall physiological functions [132]. Acetate, the most prevalent SCFA, is used by peripheral tissues for energy and helps regulate cholesterol levels [133]. Propionate, though less abundant, aids in glucose regulation and is linked to a lower risk of metabolic disorders such as obesity and type 2 diabetes [134]. Butyrate is vital for colon health, serving as the primary energy source for colonocytes, enhancing gut barrier integrity through mucus production and tight junction formation and providing anti-inflammatory benefits [135,136]. SCFAs exert their physiological effects through specific receptors on the surface of various cell types in the gut and other tissues. The primary SCFA receptors are G-protein coupled receptors (GPCRs), namely, GPR41 (also known as free fatty acid receptor 3, FFAR3), GPR43 (FFAR2), and GPR109A [137,138]. Research by Han et al. demonstrated that GLPs alleviated DSS-induced colitis in mice. The study found that an increased expression of SCFA receptors, such as GPR43, GPR109A, and the olfactory receptor Olfr78, along with elevated SCFAs levels in feces, indicated substantial SCFA absorption in the colon. This upregulation significantly enhanced the integrity of intestinal tight junctions, including zonula occludens-1, claudin-1, and mucin, thereby strengthening the intestinal barrier. Furthermore, the beneficial effects of SCFAs on the intestinal barrier may be linked to the presence of bacteria such as *Enterorhabdus*, *Desulfovibrio*, *Alistipes*, and *Bacteroides acidifaciens* [139]. SCFAs are also vital in regulating the immune response, maintaining immune tolerance, and preventing excessive inflammation [140,141]. GLPs can alleviate DSS-induced colitis by increasing SCFAs levels. Additionally, GLP treatment resulted in decreased levels of CC chemokine ligand-25 (CCL-25) and CC-chemokine receptor-9 (CCR-9), while CD40 and TGF-β1 levels were elevated [142]. CCR-9, the receptor for CCL-25, is primarily expressed on T cells that migrate to the gut. By inhibiting CCL-25 and CCR-9, GLPs reduce the infiltration of inflammatory T cells into the gut, thereby mitigating intestinal inflammation [143,144]. CD40, a co-stimulatory protein on antigen-presenting cells, interacts with the CD40 ligand on T cells to activate and proliferate both T cells and B cells, thus enhancing adaptive immunity [145].

**Table 1:** Biological activities of GLPs, related mechanisms, and structural information.

Bioactivities	Mechanism	Ref.
Antioxidant	Radical (hydroxyl, DPPH, and ABTS) scavenging capacity, regulation of antioxidant enzyme levels (MDA, SOD) in HK-2 cells, and decreases ROS levels.	[146]
Antioxidant	Radical (ABTS, hydroxyl, and nitrite) scavenging capacity.	[22]
Antioxidant	Decreases senescence-associated β-galactosidase activity and suppression of p21 and p53 gene expression.	[72]
Antitumor	Inhibition of tumor cell proliferation in vitro through the apoptosis-related Fas/FasL signaling pathway.	[62]
Antitumor	Inhibition of tumor cell proliferation in vitro, enhancement of NK cell activity, and increases levels of serum cytokines in vivo.	[106]
Hypoglycemic	Regulation of blood sugar levels. Increases in SOD, GSH-Px, and total antioxidant capacity.	[73]
Hypoglycemic	Inhibition of α-glucosidase activity.	[147]
Intestinal health	Modulation of gut microbiota and increases short-chain fatty acids.	[142]
Intestinal health	Modulates gut microbiota, increases short-chain fatty acids, and enhances the expression of tight junction proteins and MUC-2.	[17,139]
Anti-influenza virus	Inhibits viral replication and decreases viral adsorption ability.	[148]
Wound healing	Promotes cell proliferation and migration through activation of the PI3K/aPKC signaling pathway. Enhances epithelial layer thickness and collagen deposition in vivo.	[57]

## 6. Conclusions

GLPs are a promising focus of research due to their diverse and potent biological activities. This review underscores the significant progress made in extracting and characterizing GLPs. Traditional thermal extraction methods have been pivotal for isolating these polysaccharides, but advanced techniques such as UAE, MAE, and EAE have greatly enhanced yield and extraction efficiency. The structural analysis of GLPs, using techniques like HPLC, GC, and NMR spectroscopy, has provided detailed insights into their monosaccharide composition, molecular weight, and structural configurations.

The biological activities of GLPs are well documented, with strong evidence supporting their antioxidant, immunomodulatory, antitumor, and intestinal health properties. These bioactivities underscore the potential applications of GLPs in health and disease management. Their antioxidant properties can help prevent oxidative stress-related diseases, while their anti-inflammatory effects may offer relief for conditions such as inflammatory bowel disease and ulcerative colitis. The antitumor activities of GLPs, including their ability to induce apoptosis, inhibit tumor cell proliferation, and modulate immune responses, highlighting their potential in cancer therapy. Additionally, the prebiotic effects of GLPs on gut microbiota suggest their potential in improving gut health and managing metabolic disorders.

Future research should focus on several key areas to fully harness the therapeutic potential of GLPs. Clinical trials are essential to validate the health benefits observed in preclinical studies and establish effective dosages for human consumption. Mechanistic studies are needed to elucidate the pathways through which GLPs exert their biological effects, aiding in the development of targeted therapies. Moreover, exploring the synergistic effects of GLPs with other bioactive compounds could lead to the development of more effective functional foods and nutraceuticals. Investigating the sustainability and scalability of GLP production will also be crucial for their widespread application. Overall, continued research and development in this field hold great promise for the integration of GLPs into health and wellness products, contributing to the prevention and treatment of various diseases.

## Data Availability

No new data were created or analyzed in this study. Data sharing is not applicable to this article.
